# Sea Anemone *Stichodactyla Haddoni* Venom: Extraction Method Dictates Composition and Functional Potency

**DOI:** 10.3390/md23090333

**Published:** 2025-08-23

**Authors:** Meiling Huang, Ming Li, Rong Zhu, Kailin Mao, Kun Pan, Xuefeidan Liu, Bingmiao Gao

**Affiliations:** Hainan Key Laboratory for Research and Development of Tropical Herbs, Engineering Research Center of Tropical Medicine Innovation and Transformation of Ministry of Education, School of Pharmacy, Hainan Medical University, Haikou 571199, China

**Keywords:** sea anemone, *Stichodactyla haddoni*, venom extraction, functional proteomics, cytotoxicity, insecticidal activity

## Abstract

Sea anemone venoms contain diverse toxins that have significant pharmacological potential, including anticancer, ecticidal, and immunotherapeutic properties. However, critically, the extraction methodology influences venom composition and bioactivity. This study characterized venom from *Stichodactyla haddoni* obtained via homogenization, electrical stimulation, and milking. Extraction yields varied significantly between methods: the homogenization, electrical stimulation, and milking of healthy sea anemones yielded crude venoms at rates of 17.8%, 3.4%, and 1.5%, respectively. SDS-PAGE revealed distinct protein banding patterns and concentrations, while RP-HPLC demonstrated method-dependent compositional differences. Comprehensive proteomic profiling identified 2370 proteins, encompassing both unique and shared components across extraction techniques. Label-free quantitative analysis confirmed significant variations in protein abundance that was attributable to the extraction method. Cytotoxicity assays against cancer cell lines revealed concentration-dependent inhibition, with milking-derived venom exhibiting the highest potency. Insecticidal activity against *Tenebrio molitor* was also method-dependent, with milking venom inducing the highest mortality rate. These findings elucidate the profound impact of extraction methodology on the protein composition and functional activities of *S. haddoni* venom, providing crucial insights for its optimized exploitation in pharmacological development.

## 1. Introduction

Sea anemones represent an abundant marine resource, with over a thousand species distributed worldwide. Among them, *Stichodactyla haddoni* grows on rocky as well as coral substrate and is widely distributed in the tropical and subtropical waters of the Indo-Pacific area, from Mauritius to Fiji, and from the Ryukyu islands of southern Japan to Australia, including abundant populations in the South China Sea [1]. Sea anemones pose a major threat to swimmers, and stings can cause severe symptoms such as itching, edema, muscle aches, hemolysis, organ failure, and even death [2,3,4]. Sea anemone venom, primarily found in the nematocysts of tentacles, is a complex mixture of many bioactive molecules. It has been reported to contain several types of biologically active proteins and peptides, including phospholipase A2 enzymes (PLA2), cytolysins, peptide neurotoxins, enzyme inhibitors, and insecticidal components [5,6,7]. However, these bioactive components are usually unstable and can be easily degraded or even completely lost due to factors such as enzymatic degradation, high temperatures, extreme pH, and the presence of metal ions [8]. Therefore, isolating bioactive components from venom is challenging, especially given the fact that different venom-extraction methods lead to differences in the products extracted, making the exploration of efficient means for isolating the bioactive biomolecules from anemone venom a valuable endeavor.

Research on the venom of the sea anemone *S. haddoni* remains limited [9,10]. Five proteins have been isolated and partially characterized from its nematocyst venom: SHTX-I/SHTX-II, SHTX-III, SHTX-IV, SHTX-V, and SHTX-K [9]. SHTX-I/SHTX-II is a neurotoxin targeting voltage-gated potassium channels, inhibiting α-dendrotoxin binding to synaptic membranes (IC_50_ = 270 nM) [9]. SHTX-III, a Kunitz-type protein, inhibits serine proteases and blocks voltage-gated potassium channels. SHTX-IV is a β-defensin-like protein that specifically binds voltage-gated sodium channels (Nav), delaying their inactivation. While β-defensins typically function as immune proteins with broad-spectrum antimicrobial activity, anemone toxins like SHTX-IV uniquely target ion channels [11,12,13]. SHTX-V is an EGF-like protein; EGF toxins, first identified in Stichodactyla gigantea, cause crab paralysis [14]. Based on sequence similarity, SHTX-K is predicted to block Kv1.1/KCNA1 and Kv1.3/KCNA3 channels [15,16].

Interest in sea-anemone-derived bioactive peptides has grown significantly in recent decades due to their unique structures and potential as nervous-system-targeting drug candidates [17,18,19]. High-quality crude venom is essential for advancing both research and drug development. Toxins have been obtained using various methods: milking yielded hemolytic toxins HMG I and HMG II from *Heteractis magnifica*, while homogenization isolated β-defensin toxins RP II and RP III [20,21]. Homogenization and milking isolated the toxins UCI and UcPLA2, respectively, from Urticina crassicornis [22,23]. Electrical stimulation isolated BcgIII, a potential Kv channel blocker, from *Bunodosoma caissarum* [24,25]. Crucially, venom toxicity varies with extraction method, impacting subsequent toxin studies. However, comparative studies on the venoms extracted by different methods from the same anemone species are lacking.

This study directly compares venoms obtained using different extraction techniques, focusing on compositional differences and their effects on biological activity. We analyzed protein content and composition via high-performance liquid chromatography combined with ultraviolet spectrophotometry (HPLC-UV), sodium dodecyl sulfate–polyacrylamide gel electrophoresis (SDS-PAGE), and proteomic analysis. We also evaluated the anticancer and insecticidal activities of the venoms extracted by each method. Our findings provide a scientific basis for selecting appropriate venom-extraction techniques, highlighting how methodology influences venom composition and activity. This work offers methodological support and a technical reference for novel venom research, significantly advancing the exploration of venom’s medicinal potential.

## 2. Results

### 2.1. Venom Protein Characteristics

**Figure 1:**
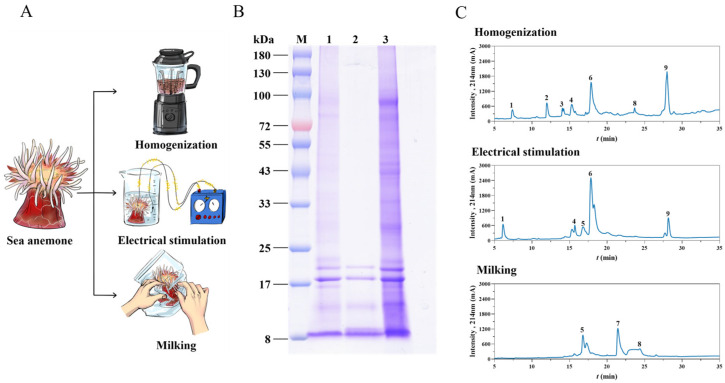
Extraction and analysis of crude venom from *S. haddoni* using three different methods. (**A**) Schematic of venom-extraction techniques. (**B**) SDS-PAGE analysis under reducing conditions (12% gel). Lane M: Molecular weight marker; Lane 1: Crude venom extract (milking); Lane 2: Crude venom extract (electrical stimulation); Lane 3: Crude venom extract (homogenization). (**C**) Reverse-phase HPLC chromatograms (C18 column) of crude venom extracts.

Crude venom extracts from three healthy sea anemone *S. haddoni* were obtained via homogenization, electrical stimulation, and milking (the total sea anemone mass measured before each experiment was 20.78 g, 24.62 g, and 22.54 g), respectively (Figure 1A). The protein concentrations of the supernatants obtained by these methods, determined using a BCA assay kit, were 16.5 mg/mL (homogenization), 0.939 mg/mL (electrical stimulation), and 1.697 mg/mL (milking). The corresponding crude venom yields were 6270 mg (301.7 mg/g sea anemone mass), 272.31 mg (11.06 mg/g sea anemone mass), and 315.64 mg (14.0 mg/g sea anemone mass).

Venom composition was assessed using SDS-PAGE (Figure 1B) and HPLC-UV. SDS-PAGE revealed distinct protein banding patterns across all extraction methods within the 8–100 kDa range. Common bands were consistently observed between 8 and 20 kDa. Notably, the venom obtained through electrical stimulation lacked detectable bands >25 kDa, while both venoms harvested via milking and homogenization exhibited higher molecular weight bands (>25 kDa), with homogenization extraction being associated with the most intense staining. HPLC separation (Figure 1C) demonstrated significant compositional differences: homogenization venom resolved into nine distinct fractions (F1–F9), electrical stimulation yielded five fractions (F1, F4, F5, F6, F9), and milking produced only three fractions (F5, F7, F8).

### 2.2. Protein Identification Statistics

**Figure 2:**
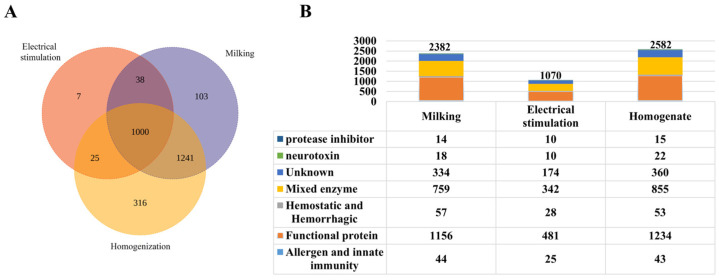
Proteomic characterization of *S. haddoni* venom extracted by different methods. (**A**) Venn diagram of protein identification (trypsin digestion/LC-MS/MS) showing unique and shared proteins across extraction methods. (**B**) Functional classification: A stacked bar graph showing the proportional distribution of toxin categories; a table enumerating proteins per functional category.

Using a proteomic database derived from *S. haddoni* transcriptomic data, we identified 2730 proteins across the three venom-extraction methods used in this study. Distribution analysis revealed 103 proteins exclusive to milking venom, 316 to homogenization venom, and 7 to electrical stimulation venom, with 1000 proteins common to all three methods. Pairwise overlaps included 25 proteins shared between electrostimulation and homogenization, 38 between electrical stimulation and milking, and 1241 between milking and homogenization (Figure 2A). Functionally classified via InterPro, proteins were grouped into seven primary categories: protease inhibitors, neurotoxins, mixed enzymes, hemostatic and hemorrhagic proteins, functional proteins, allergen and innate immunity proteins, and unknown proteins (Figure 2B). Notably, milking yielded 2382 proteins, including 18 neurotoxins (0.76%); electrical stimulation yielded 1070 proteins, including 10 neurotoxins (0.93%); and homogenization yielded 2582 proteins, including 22 neurotoxins (0.85%).

### 2.3. Differential Protein Abundance Analysis

**Figure 3:**
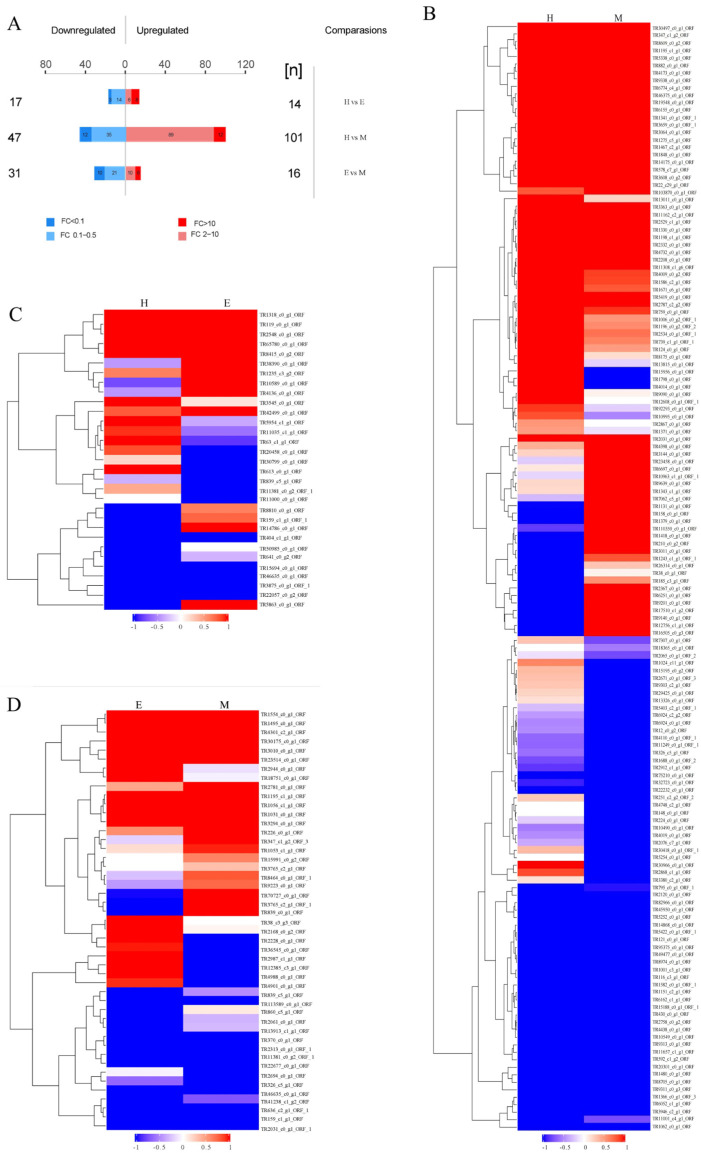
Comparisons of proteomic analysis of *S. haddoni* venoms extracted using different methods. (**A**) Differential abundance statistics of upregulated/downregulated proteins. (**B**–**D**) Pairwise hierarchical clustering heatmaps: (**B**) homogenization vs. milking; (**C**) electrical stimulation vs. homogenization; (**D**) electrical stimulation vs. milking. Color gradient: red (upregulation); blue (downregulation); gray undetected proteins. (M: milking; E: electrical stimulation; H: homogenization).

Label-free quantitative proteomics (LFQ) was employed to compare protein abundance profiles across the venoms obtained by milking, electrical stimulation, and homogenization. Hierarchical clustering heatmaps (Figure 3) visualize relationships between venom proteomes. Quantitative analysis identified 226 differentially abundant proteins (log FC ≥ 2 or log FC ≤ −2 and *p*-value < 0.01 or FDR ≤ 0.05), with significant differences observed in electrical stimulation vs. homogenization: 31 proteins; electrical stimulation vs. milking: 47 proteins; and milking vs. homogenization: 148 proteins (Figure 3A). Functional annotation via InterPro structural domains (after redundancy filtering) classified 131 of these differentially expressed proteins as upregulated and 95 as downregulated. The most substantial abundance variations occurred between the milking-and homogenization-derived venoms. Clustering analysis revealed distinct abundance patterns, with pronounced separation between electrical-stimulation-derived proteomes and those obtained via milking or homogenization (Figure 3B–D). The most substantial differences in abundance occurred between the venoms extracted through milking and homogenization.

### 2.4. Cytotoxicity Assessment of Crude Venom

**Figure 4:**
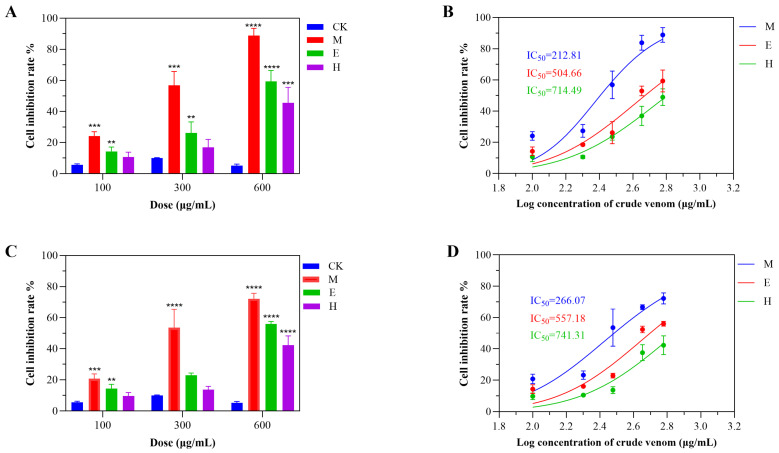
Method-dependent cytotoxicity profiles of *S. haddoni* crude venoms. (**A**) Inhibition of A549 cell viability (24 h exposure). (**B**) IC_50_ values for A549 cytotoxicity. (**C**) Inhibition of HeLa cell viability (24 h exposure). (**D**) IC_50_ values for HeLa cytotoxicity. Data: Means ± SD (*n* = 3); significance: ** *p* < 0.01, *** *p* < 0.001, **** *p* < 0.0001 vs. control. Abbreviations: CK (control), M (milking), H (homogenization), E (electrical stimulation).

Evaluation of the cytotoxic activity of *S. haddoni* venoms extracted via milking (M), electrical stimulation (E), and homogenization (H) revealed significant method-dependent bioactivity against A549 and HeLa cancer cell lines. All venoms exhibited concentration-dependent toxicity (100–600 μg/mL, 24 h exposure), with a consistent potency ranking of M > E > H (Figure 4). For A549 cells, M venom induced 24.1%, 56.8%, and 88.9% growth inhibition at 100, 300, and 600 μg/mL, respectively; the same values for the other extraction methods were 10.7%, 17.0%, and 45.6% (H) and 14.3%, 26.2%, and 59.3% (E) (Figure 4A). The corresponding IC_50_ values were 212.81 μg/mL (M), 714.49 μg/mL (H), and 504.66 μg/mL (ES) (Figure 4B). Similarly, HeLa cell inhibition reached 20.8%, 53.5%, and 72.2% for M, 9.7%, 13.7%, and 42.3% for H, and 14.3%, 22.9%, and 56.1% for E (Figure 4C), with IC_50_ values of 266.07 μg/mL (M), 741.31 μg/mL (H), and 557.18 μg/mL (E) (Figure 4D). Critically, M venom demonstrated significant cytotoxicity even at low concentrations (100 μg/mL), whereas the concentration of the H and E venoms needed to be ≥300 μg/mL for substantial effects. These data conclusively establish that the extraction methodology dictates venom cytotoxicity profiles.

### 2.5. Insecticidal Activity of Crude Venom

**Figure 5:**
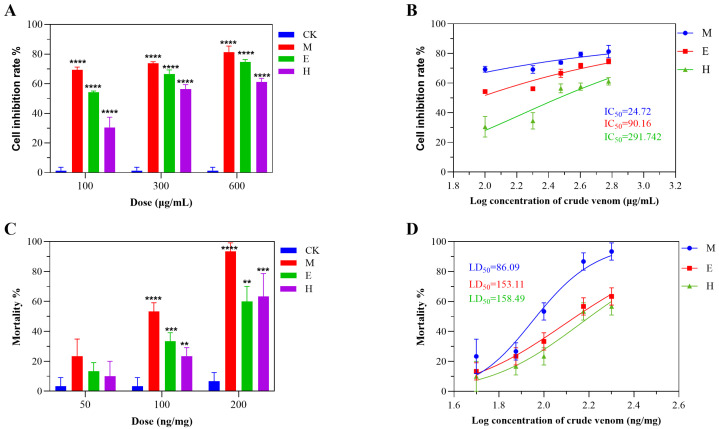
Insecticidal activity of *S. haddoni* crude venoms extracted via different methods. (**A**) Sf9 (Spodoptera frugiperda) cell viability inhibition (24 h). (**B**) IC_50_ values for Sf9 cytotoxicity. (**C**) *Tenebrio molitor* mortality (24 h post-injection). (**D**) LD_50_ values for *T. molitor* toxicity. Data: Means ± SD (*n* = 3 technical replicates); significance: ** *p* < 0.01, *** *p* < 0.001, **** *p* < 0.0001 vs. control. Abbreviations: CK (control), M (milking), H (homogenization), E (electrical stimulation).

The insecticidal potential of *S. haddoni* crude venoms was assessed through Sf9 cell cytotoxicity and *Tenebrio molitor* bioassays. The venoms obtained through the three extraction methods (milking, electrical stimulation, and homogenization) all demonstrated significant Sf9 cell toxicity, even at low concentrations. The venoms from milking and electrical stimulation exhibited the greatest (non-concentration-dependent) inhibition, while homogenization-derived venom plateaued at medium–high doses, yielding IC_50_ values of 24.72 μg/mL (M), 90.16 μg/mL (E), and 291.74 μg/mL (H) (Figure 5A,B). Intraperitoneal injection in *T. molitor* (n = 10/group) revealed method-dependent lethality: LD_50_ values were 86.09 ng/mg (M), 153.11 ng/mg (E), and 158.49 ng/mg (H) (Figure 5D). All venoms induced neurotoxic symptoms (abdominal spasms, paralysis), with mortality increasing in a dose-dependent manner (M > E ≥ H). Milking-derived venom induced the highest levels of mortality across concentrations (Figure 5C), while E venom showed greater efficacy than H venom at low–medium doses, but this phenomenon reversed at high doses. Control tests revealed no toxicity, confirming that the venoms’ insecticidal potential correlates with the extraction methodology.

## 3. Discussion

The venom produced by sea anemones has a wide scope for medicinal development, and the extraction of sea anemone venom is important for its research and development. However, there is a lack of information on the best method for the extraction of crude sea anemone venom. There is also no systematic comparison of the venoms extracted through different techniques. In this study, to investigate different methods for obtaining sea anemone venom, we combined proteomics with traditional activity studies to compare venoms extracted via three different methods. Homogenized venom showed higher toxin complexity compared to electrical-stimulation-derived and milked venoms (Figure 2A). Surprisingly, the venom obtained via milking showed the greatest biological activity. The homogenization method was shown to be more effective than both electrical stimulation and milking methods for extracting proteins from the crude venom of *S. haddoni* sea anemones. Based on RP-HPLC analysis, the crude venom extracted via the homogenization method contained seven major components. By contrast, the milking method produced venom with only three main components. SDS-PAGE analysis indicated that homogenization and milking produced crude venom with similar band distributions. The crude venom obtained through milking exhibited prominent small-molecule peptide bands but had large-molecule protein bands that were significantly weaker in intensity than those in homogenization-derived venom. In contrast, crude venom obtained via electrical stimulated only exhibited strong bands at 2.7 kDa and 66 kDa and a weak band at 4.1 kDa. In order to further study the components of *S. haddoni* venoms extract via different methods, trypsin digestion was performed on the different venom samples, while LC-MS/MS analysis was performed on the tims TOF Pro mass spectrometer. This approach facilitated both the qualitative and quantitative analyses of three venom samples. Following the processing of the resulting MS/MS spectra, 2382, 1070, and 2582 proteins were identified in the *S. haddoni* venom obtained through the milking, electrical stimulation, and homogenization methods, respectively. Overall, different extraction methods are associated with differences in the number of and types of proteins; this will have an impact on the subsequent discovery of functional drugs from anemone venom.

Sea anemone venom has been reported to have multiple biological effects, including anticancer and insecticidal properties [18,26,27,28]. Previous reports suggest that the crude venom of *S. haddoni* exhibits notable anticancer activity [28,29]. It is widely acknowledged that the IC_50_ of sea anemone crude venom can vary, even when extracted using a single method. For example, crude venom from *Heteractis magnifica* sea anemone, obtained by the milking method, exhibited different inhibitory effects on A549 cells and T47D cells in different studies [30,31]. In our current study, three different extraction methods (milking, electrical stimulation, and homogenization) were used to obtain crude venom, which was subsequently evaluated for its inhibitory effects against A549 and HeLa cells. While homogenization yielded the highest total protein content (2582 proteins) and neurotoxin count (22 neurotoxins), electrical stimulation demonstrated the highest neurotoxin extraction efficiency (0.93% of total proteins) despite yielding the lowest overall protein quantity (1070 proteins). Interestingly, neurotoxins were predominantly classified within the ShK family, suggesting that toxin family composition may determine venom bioactivity. Our data revealed significant extraction-method-associated differences in the inhibitory effects of these crude venom extracts. Interestingly, the crude venom obtained through milking exhibited the highest antiproliferative efficacy, with double the inhibitory effect observed when using venoms obtained using the electrical stimulation and homogenization techniques. The homogenization method produced the weakest toxicity against cancer cells, displaying relatively low inhibitory activity when used at the same concentration as the venoms obtained via the other methods.

The same pattern was observed for venoms extracted by all three methods in insecticidal activity studies. Therefore, we conclude that extraction methodology fundamentally shapes venom bioactivity through differential neurotoxin recovery. While all venoms showed dose-dependent toxicity in mealworm larvae, milking-derived venom consistently exhibited the strongest potency across models—attributable to its superior neurotoxin concentration per biomass, higher than electrical stimulation despite homogenization’s higher absolute neurotoxin yield. This neurotoxin density directly explains milking’s dominant efficacy (lowest LD_50_ 86.09 ng/mg in mealworms; IC_50_ 24.72 µg/mL in Sf9). Conversely, homogenization’s coextracted structural proteins caused functional dilution, requiring significantly higher venom concentrations to achieve significant effects despite high neurotoxin content. This dilution also explains the concentration-dependent reversal between methods: electrical stimulation surpassed homogenization at low doses (higher neurotoxin proportion: 0.93% vs. 0.85%) but was overtaken at high concentrations where homogenization’s mass delivery compensated for dilution. Critically, neurotoxin recovery per gram tissue (not total protein) determines insecticidal efficacy. Milking optimizes this parameter while preserving functional integrity, making it ideal for applications demanding high specific toxicity.

The observed variation in composition and toxicity among the sea anemone crude venom samples can be attributed to a primary factor. Differences in the purity of the crude venom and the presence of high-molecular-weight compounds with low toxicity, such as receptor proteins and enzyme proteins, may contribute to these variations [32]. In venom activity experiments, the venom extracted via the milking method demonstrated the highest activity. The activity of venom extracted via homogenization exhibited concentration-dependent activity. Proteomic analysis revealed that homogenization-derived venom contained more toxin proteins; however, it should be noted that compared to milking- and electrical-stimulation-derived venoms, it also contains considerably more non-toxic proteins. Consequently, the activity of homogenization-extracted venom at lower concentrations is weaker, but as the concentration increases, so does its activity. In the proteome of the milking-extracted venom, a total of 18 types of toxin sequences were identified, and their activity was minimally affected by non-toxic proteins. Because of this, the venom extracted via milking displayed higher activity than the venom extracted using the other two methods. A total of 10 venom proteins were identified in the electrical-stimulation-extracted venom. Although non-venom proteins were less abundant in this venom than in the milking and homogenization-derived venoms, toxin proteins were also significantly less abundant. In comparison with homogenization, milking emerges as a more effective method for extracting high-activity sea anemone crude venom. However, the homogenization method can yield a greater variety of sea anemone toxins. In summary, these findings underscore the importance of the extraction method used for sea anemone venom in determining its subsequent efficiency and activity.

It is worth noting that the homogenization method allows for the extraction of more toxins. However, it must be noted that this method can only extract anemone venom in an unsustainable and non-reproducible manner. Compared with the other methods, the results for toxin extraction via electrical stimulation are relatively poor, mainly due to the fact that the high voltage and current of the electrical stimulation process can cause severe damage or even death to the anemones, as well as lead to alteration of anemone toxins. The milking method gives rapid access to highly active venom, which can be sustainably extracted from the same anemone. However, proteomic studies showed that the amount of toxin obtained using the milking method is lower than that when using the homogenization method. It is also worth noting that the yield of toxin obtained by the milking method is relatively low. These considerations highlight the need to carefully evaluate the advantages and disadvantages of different venom-extraction methods in order to select the appropriate extraction method for venom research. The results of this study reveal that the method of extraction has a significant effect on the crude toxins obtained from sea anemones. Understanding these distinctions is essential to better investigate the potential applications of sea anemone crude toxins in the field of pharmacological research and development.

## 4. Materials and Methods

### 4.1. Sample Preparation

*S. haddoni* sea anemone specimens were collected from the Xisha islands, Hainan Province, China. They were transported using polythene bags and maintained alive in laboratory conditions. Healthy sea anemones were selected for experiments.

### 4.2. Venom Acquisition

Homogenization: A total of three healthy anemones weighing about 20.78 g were selected for this experiment. The anemones were carefully placed on a clean bench, the tentacles were cut off with clean scissors, and the tentacle tissue from each anemone was combined in a homogenizer. Distilled water was used in the homogenization process. The sample was then centrifuged at 10,528× *g* for 20 min. The supernatant with a total volume of 375 mL was collected in centrifuge tubes [33,34].

Electrical stimulation: Three individual *S. haddoni* samples weighing a total of 24.62 g were selected from healthy growing anemones. Each anemone was taken from an aquarium, placed in a 100 mL beaker, and cleaned with tweezers and distilled water to remove contaminants. Subsequently, the anemones were immersed in a separate beaker containing 50 mL of artificial seawater and electrical stimulation was performed using two carbon electrodes (100 V, 10 ms, 20 Hz, for 60 s). After the stimulation of each sea anemone, the solution was filtered through gauze and filter paper to remove body particles, including discharged nematocyst capsules, and the supernatant with a total volume of 290 mL was then collected and combined in one centrifuge tube [35,36].

Milking: Three individual healthy *S. haddoni* samples weighing a total of 22.54 g were transferred from the aquarium to a clean plastic bag, and the sea anemone tentacles were gently massaged with fingers to obtain crude venom. After the massaging of each anemone, the solution was filtered through gauze and filter paper to remove body particles, including discharged nematocyst capsules; the supernatant with a total volume of 186 mL was collected and combined in one centrifuge tube [18,37,38].

### 4.3. SDS–PAGE Analysis

Crude toxin extract was dissolved in ddH_2_O to a concentration of 1 mg/mL. The solution was centrifuged at 1360× *g* for 10 min to pellet insoluble material. Subsequently, 10 μL of supernatant was mixed with 190 μL of BCA working reagent (Biosharp, Hefei, China) and incubated at 37 °C for 30 min. Absorbance at 562 nm was measured using a microplate reader. The crude venom was analyzed via SDS–PAGE according to the Laemmli and Amersham Pharmacia [39,40]. Based on the BCA measurement results, 1 mg/mL of protein was mixed with 2 × loading buffer containing β-mercaptoethanol (*v*/*v* = 1:1), boiled for 10 min, and then separated using 5% concentrated gel and 12% separation gel. The proteins were stained with Coomassie Brilliant Blue R-250 (Yeasen Biotechnology, Shanghai, China), and the molecular weights were determined by comparison with standard proteins.

### 4.4. RP-HPLC Analysis

The sea anemone toxins extracted using different methods were analyzed on the Shi-madzu system using a 0.46 × 15 cm Shimadzu pack GIST C18 column (Shimadzu Corporation, Kyoto, Japan). Solvents A and B contained 0.1% TFA in HPLC-grade water and HPLC-grade acetonitrile, respectively. The column was initially equilibrated with 5% acetonitrile at room temperature. The toxin was filtered through 0.45 μm syringe filters, and the injection volume was 100 μL. Samples containing 10 mg crude toxin in 1 mL of solution were analyzed by applying a linear gradient of 5–70% acetonitrile over 65 min at 1 mL/min. The chromatographically separated compounds were detected with UV at a wavelength of 214 nm.

### 4.5. RNA Extraction, mRNA Library Synthesis, and Illumina Sequencing

The tentacles and columnar tissue of *S. haddoni* were mixed, frozen in liquid nitrogen, and then transported to the sequencing laboratory for transcriptome sequencing. Total RNA was extracted from *S. haddoni* tissue using TRIzol^®^ Reagent (Thermo Fisher Scientific, Waltham, MA, USA) according to the manufacturer’s instructions. The Nanodrop ND-2000 system was used to evaluate the purity of RNA samples in terms of the A260/A280 absorbance ratio (Thermo Scientific, Waltham, MA, USA). The RIN of the RNA was determined using an Agilent Bioanalyzer 4150 system (Agilent Technologies, Santa Clara, CA, USA). Paired-end libraries were prepared using an ABclonal mRNAseq Lib Prep Kit (ABclonal, Wuhan, China) following the manufacturer’s instructions. The mRNA was isolated from 1 μg total RNA through oligo(dT) magnetic bead purification, followed by fragmentation, which was achieved using divalent cations at elevated temperatures in ABclonal First-Strand Synthesis Reaction Buffer. Subsequently, first-strand cDNAs were synthesized with random hexamer primers and reverse transcriptase (M-MLV) using mRNA fragments as templates, followed by second-strand cDNA synthesis using DNA polymerase I, RNAseH, buffer, and dNTPs. The synthesized double-stranded cDNA fragments were then adapter-ligated for preparation of the paired-end library. Adaptor-ligated cDNA was used for PCR amplification. PCR products were purified (AMPure XP system) before library quality was assessed on an Agilent Bioanalyzer 4150 system. Finally, the library preparations were sequenced on an Illumina Novaseq 6000 (Illumina, San Diego, CA, USA), and 150 bp paired-end reads were generated.

### 4.6. Protein Digestion

The amount of venom obtained through the three extraction methods was quantified using the BCA method. An appropriate amount of protein was taken from each sample and subjected to trypsin digestion using the filter-aided proteome preparation (FASP) method [41]. DTT (with the final concentration of 10 mM) was added to each and mixed at 600 rpm for 1.5 h (37 °C). After the samples had cooled to room temperature, IAA was added at a final concentration of 20 mM to block reduced cysteine residues, and the samples were incubated for 30 min in darkness. Next, the samples were transferred to the filters. The filters were washed with 100 μL UA buffer three times and then 100 μL 25 mM NH_4_HCO_3_ buffer twice. Finally, trypsin was added (the trypsin/protein (*wt*/*wt*) ratio was 1:50) and the samples were incubated at 37 °C for 15–18 h (overnight). The resulting peptides were collected as a filtrate. The peptides in each sample were desalted on C18 Cartridges (Empore SPE Cartridges C18 (standard density), bed I.D. 7 mm, volume 3 mL, Sigma, Livonia, MI, USA), concentrated by vacuum centrifugation, and reconstituted in 40 µL of 0.1% (*v*/*v*) formic acid (solution A).

### 4.7. Mass Spectrometric Identification

LC-MS/MS analysis was performed on a tims TOF Pro mass spectrometer (Bruker Daltonics, Billerica, MA, USA) coupled to Easy nLC (Thermo Fisher Scientific, Waltham, MA, USA,). The peptides were loaded onto a reverse-phase trap column (Thermo Scientific Acclaim PepMap100, 100 μm × 2 cm, nanoViper C18) connected to the C18 reverse-phase analytical column (Thermo Scientific Easy Column, 10 cm, 75 μm inner diameter, 3 μm) in buffer A (0.1% formic acid in water) and separated with a linear gradient of buffer B (84% acetonitrile and 0.1% formic acid) at a flow rate of 300 nL/min. The mass spectrometer was operated in positive-ion mode. MS data were acquired using a data-dependent top 20 method, dynamically choosing the most abundant precursor ions from the survey scan (300–1800 *m*/*z*) for HCD fragmentation. The automatic gain control (AGC) target was set to 1e6, and the maximum inject time to 50 ms. The dynamic exclusion duration was 30.0 s. Survey scans were acquired at a resolution of 60,000 at *m*/*z* 200 and the resolution for HCD spectra was set to 15,000 at *m*/*z* 200, and the isolation width was 1.5 *m*/*z*. The normalized collision energy was 30 eV, and the underfill ratio, which specifies the minimum percentage of the target value likely to be reached at maximum fill time, was defined as 0.1%.

### 4.8. Protein Identification

The raw data files generated by the mass spectrometer were searched in the transdecoder database built from the *S. haddoni* transcriptome, using the MaxQuant software (version 1.6.14) [42]. The research parameters used were as follows: trypsin specificity; precursor mass tolerance adjusted to ±20 ppm; and fragmentation ion mass (tolerance ± 0.05 Da). Oxidized methionine (+15.99 Da) and acetylation (+42.01 Da) were defined as variable modifications. Carbamidomethylation (+57.02) was also added as a variable modification for alkylated and reduced samples. The identified peptides were then sorted by their mean local confidence to select the best spectra for annotation and filtered using FDR ≤ 1%.

### 4.9. Quantitative Data Analysis

Label-free quantitative (LFQ) analysis utilizes protein identification results obtained through insolution tryptic digestion and LC-MS/MS and performs relative quantitative analysis of proteins based on the integrated area of ion peak intensity detected by mass spectrometry. MaxQuant software (version 1.6.14) was employed for data analysis. In the analysis, LFQ intensities were normalized and consideration was given to unique and razor peptides, with the minimal ratio count set to 1. Before the analysis, the contaminants and reverse identifications were removed and the values of LFQ intensities were transformed to log2. Significance B was applied exclusively to protein groups that were quantified consistently across all technical replicates within the three groups (homogenization, electrical stimulation, and milking extraction). Fold-change values for protein groups showing statistical significance between samples were calculated using LFQ intensities.

### 4.10. Cell Line and Cell Culture

The human cell lines HeLa (cervical carcinoma) and A549 (lung cancer) and the Spodoptera frugiperda sf9 cell line were obtained from Hainan Provincial Key Laboratory of Carcinogenesis and Intervention. SF9 cells were grown in Sf-900 II SFM medium containing 10% fetal bovine serum (Gibco, Livonia, MI, USA,) and 1% penicillin/streptomycin (Gibco), while the HeLa and A549 cell lines were grown in DMEM medium containing 10% fetal bovine serum (Gibco) and 1% penicillin/streptomycin (Gibco). SF9 cells were cultured in a fully humidified incubator at 27 °C, while HeLa and A549 cell lines were incubated in a humidified incubator at 37 °C with 5% CO_2_.

### 4.11. Cytotoxicity Assay

The cytotoxicity of crude toxin samples obtained via different extraction methods was evaluated using cell-counting kit-8 [43]. The human non-small-cell lung cancer cell line A549, the human cervical carcinoma cell line HeLa, and the Spodoptera frugiperda cell SF9 (1 × 10^4^ per well) were incubated in 96-well plates and cultured for 4 h to allow adherence. Then, the cells were exposed to 100 μg/mL, 200 μg/mL, and 400 μg/mL sea anemone crude venom for 24 h at 37 °C in a 5% CO_2_ incubator. Cells were then washed twice with PBS and 10 μL CCK-8 solution (5 mg/mL in the medium) was added. The plate was further incubated at 37 °C for 4 h in a humidified incubator with 5% CO_2_. The plates were placed in a 550-type microplate reader and the absorption at 450 nm was measured.

### 4.12. Lethality Assays

*Tenebrio molitor* larvae (body weight 90–100 mg) were purchased from a breeding facility in Haikou, Hainan province of China. Amounts of 50 ng/mL, 100 ng/mL, and 200 ng/mL of 4 μL of crude sea anemone venom were diluted in insect physiological saline (0.7% NaCl) and injected into the abdomen of *Tenebrio molitor*. The insects injected with saline alone served as a negative control. After the injection, the responses of the *tenebrio molitor* were observed and the median lethal dose (LD_50_) was calculated.

## 5. Conclusions

This study demonstrates that the method of extraction significantly impacts the yield, composition, and bioactivity of *Stichodactyla haddoni* venom. Integrating proteomic analysis with functional assays revealed distinct profiles. Homogenization yielded the highest quantity and proteomic diversity but was associated with a substantial quantity of non-active proteins. Milking produced venom with superior functional potency in cytotoxicity and insecticidal assays despite a lower overall yield. Crucially, milking facilitates sustainable venom collection without sacrificing the anemone. These findings highlight the critical impact of extraction methodology on venom properties, providing essential guidance for optimizing the isolation of highly active toxins for marine drug discovery.

## Data Availability

All the data generated in this study, including the original files of the proteome and transcriptome, have been published online. Specifically, the proteome dataset and atlas of *Stichodactyla haddoni* are stored in iProX with login number IPX0008890000. The RNA-seq data for *Stichodactyla haddoni* are accessible in SRA with accession number PRJNA1116309.
